# Beyond the finite pool of worry: War experiences and climate change concerns in Ukraine

**DOI:** 10.1007/s13280-025-02291-w

**Published:** 2025-11-15

**Authors:** Mateusz Błaszczyk, Berenika Dyczek

**Affiliations:** Institute of Sociology, University of Wrocław, Ul. Koszarowa 3, 51-148 Wrocław, Poland

**Keywords:** Climate change perception, Finite pool of worry, Risk awareness, Russian–Ukrainian conflict, Wartime exposure

## Abstract

This study explores the relationship between self-reported war impacts on personal lives and climate change concerns in Ukraine, challenging the "finite pool of worry" hypothesis. Based on survey data, the research reveals that individuals who perceive their lives as critically affected by the war exhibit heightened climate change concerns, suggesting that severe crises can amplify awareness of interconnected global risks. The findings contribute to a broader understanding of risk perception in societies navigating multiple crises and emphasize the importance of environmental considerations in post-conflict dialogue and reconstruction efforts. The study underscores the complex social dynamics of risk perception, advocating for a shift beyond individual psychological explanations toward a more comprehensive understanding of how societies collectively navigate interconnected threats.

**Supplementary Information:**

The online version contains supplementary material available at 10.1007/s13280-025-02291-w.

## Introduction

In a world where global threats are constantly competing for our attention, how do we react when the reality of war invades our daily lives? Does the direct experience of armed conflict change our perception of other seemingly distant threats, such as climate change? The Russian invasion of Ukraine in 2022 provides a unique opportunity to address these questions. This conflict represents the first major war in a society with established climate awareness since the post-Cold War international order, offering an unprecedented empirical context to examine how direct war experiences affect concerns about long-term environmental challenges.

It is posited that war and climate change represent distinct sources of anxiety, differing in immediacy, tangibility, and personal significance. Armed conflict poses a direct, intense, and personally significant threat to individual survival and well-being. Climate change, in contrast, is often perceived as a more gradual, complex, and psychologically distant process affecting broader systems. Moreover, climate change represents a distinctive category of global risk requiring unprecedented international cooperation, irreversible consequences spanning generations, and fundamental transformation of economic systems—characteristics that distinguish it from other trauma-affected risk perceptions.

One influential framework for understanding how individuals prioritize multiple threats is the finite pool of worry (FPW) hypothesis (Weber 2006). It posits that people have a limited capacity for concern, and that acute, immediate threats may crowd out attention to more distant or abstract risks. This concept has been widely used in studies examining how crises—such as economic downturns, pandemics, or natural disasters—affect perceptions of climate change. However, findings have been mixed, suggesting that the FPW mechanism may not operate uniformly across contexts.

Recent empirical research reveals complex relationships between crisis experiences and environmental risk perceptions. Large-scale survey experiments show that framing around concurrent crises can divert attention from climate concerns: Genschel et al. (2025) found that mentioning COVID-19 or Russian military aggression reduced support for climate policies among European populations not directly affected by these events, compared to climate-focused messaging alone. However, other studies indicate that individuals can process multiple risk types simultaneously, with the temporal dimension of risks—future existential versus present-day threats—determining different types of engagement (Uysal et al. 2025). Cultural frameworks also mediate these relationships, as worldviews significantly shape how individuals perceive and respond to climate risks (Xue et al. 2016). Despite this growing research on crisis–climate interactions, a critical gap remains: existing studies examine indirect exposure or aggregate attitudes among populations not directly experiencing warfare. How direct, personal experiences of armed conflict—among populations living under active warfare—influence individual climate change concerns remains largely unexplored.

Research on the FPW hypothesis has been conducted in various contexts. Hansen et al. (2004) studied Argentine farmers, finding that increased climate change concerns were associated with decreased political concerns. Whitmarsh (2011) and Scruggs and Benegal (2012) analyzed the impact of economic crises on climate change perceptions in the UK and USA, respectively, finding some support for the FPW hypothesis—economic recession seemed to decrease concerns about climate change. Nakayachi et al. (2015) studied the effect of the 2011 earthquake and nuclear disaster in Japan on the perception of various threats, observing that increased concerns about some threats were associated with decreased concerns about others.

Researchers have paid particular attention to the impact of the COVID-19 pandemic on perceptions of climate change, yielding mixed results. Some studies support the FPW hypothesis in this context. For instance, Smirnov and Hsieh (2022) observed a decrease in climate change discussions on social media as COVID-19 cases increased, while Gregersen et al. (2022) detected a small decrease in climate change concerns in Norway during the pandemic.

However, other studies challenge the FPW hypothesis in relation to the pandemic. Evensen et al. (2021) found no evidence of decreased climate change concerns in the UK during the pandemic. Stefkovics et al. (2024) even observed an increase in climate change concerns across multiple European countries. Adding to this complexity, Ekinci and Van Lange (2023) found that exposure to COVID-19 threats did not decrease pro-environmental behavior or intentions in the UK, and in some cases, being psychologically closer to COVID-19 was associated with increased pro-environmental behavior.

Sisco et al. (2023) provided a nuanced perspective by distinguishing between a “finite pool of attention” and a “finite pool of worry.” Their comprehensive analysis across multiple countries showed that while attention to climate change decreased during the pandemic, worry about climate change did not necessarily decrease and in some cases increased.

However, alternative theoretical frameworks suggest that severe crises may heighten rather than diminish environmental concern. Beck’s (1992) risk society theory posits that modern societies increasingly grapple with manufactured, global-scale risks that transcend traditional boundaries. Crucially, Beck argues that experiences of one type of manufactured risk can increase reflexive awareness of other systemic threats. Rather than depleting a finite cognitive resource, crisis experiences may catalyze broader "risk consciousness" that encompasses multiple, interconnected threats.

This perspective aligns with emerging polycrisis frameworks (Henig and Knight 2023), which emphasize that contemporary societies face not isolated crises but cascading, interacting emergencies. From this view, war in Ukraine is not separate from climate change but part of an interconnected web of global instabilities. Experiencing one crisis may thus heighten awareness of systemic vulnerabilities, making distant threats feel more proximate and urgent.

Psychological research provides additional support. Janoff-Bulman's (1992) "shattered assumptions" framework suggests that severe trauma fundamentally disrupts basic beliefs about safety, predictability, and control. This disruption may paradoxically heighten—rather than narrow—threat awareness across multiple domains. When war experiences shatter assumptions about systemic stability, individuals may become more attuned to other systemic vulnerabilities, including environmental risks. Rather than cognitive depletion, trauma may trigger cognitive reorganization that extends beyond immediate threats.

Importantly, war experiences in Ukraine constitute not merely individual traumas but collective trauma—a shared societal experience that shapes cultural meaning-making (Alexander 2004). Collective trauma operates at a different level than individual psychological responses, involving social processes through which communities interpret, represent, and respond to catastrophic events. When entire societies grapple with manufactured risks like war, the resulting collective consciousness may extend beyond immediate threats to encompass broader systemic vulnerabilities, including environmental risks that similarly threaten collective futures.

Research on the effects of natural hazards and extreme weather events provides insights into how direct experiences with climate-related phenomena influence perceptions of climate change. Unlike other crises, these events are closely linked to the climate system itself, potentially making their impact on climate change awareness more direct. Multiple studies have shown that individuals who have experienced floods, heatwaves, or other extreme weather events tend to perceive climate change as a more immediate and serious threat (Whitmarsh 2008; Spence et al. 2011; Demski et al. 2017; Lohmann and Kontoleon 2023). Even short-term weather anomalies, such as unusually warm days, can influence beliefs and concerns about global warming (Zaval et al. 2014).

However, it is important to note that this increased awareness and concern do not always translate into significant changes in environmental behavior or support for climate policies (Whitmarsh 2008). Meta-analyzes suggest that while the overall effect of climate-related experiences on climate change beliefs is positive, it is relatively small (Bergquist et al. 2020). This implies that even when people experience phenomena directly related to climate change, other factors such as personal values, beliefs, and emotions still play crucial roles in shaping their responses to the issue.

While research on natural hazards and climate change perceptions offers insights into direct experiences, studies on the relationship between climate change and armed conflicts provide a different perspective on such interactions (Burke et al. 2015). A substantial body of research has focused on how climate change may influence the occurrence and intensity of armed conflicts. Long-term analyses suggest that climate change has often triggered or exacerbated conflicts through ecological stress, resource scarcity, and social unrest (Zhang et al. 2007). Recent research indicates that climate factors can affect organized armed conflict within countries and potentially amplify future conflict risks (Buhaug 2015; Mach et al. 2019). Also the analysis by Schleussner et al. (2016), Ide et al. (2020) demonstrates that climate-related disasters increase the risk of conflict outbreaks, particularly in countries with ethnic exclusion, low levels of development, and large populations.

Specific mechanisms linking climate and conflict have also been identified. Research has shown that deviations from normal precipitation and temperatures can systematically increase conflict risk, possibly through impacts on agricultural production and economic performance (Hsiang et al. 2013). Strong historical relationships between civil conflict and temperature have been reported, leading to projections of increased armed conflict incidence due to climate change (Burke et al. 2009).

Research shows that armed conflicts contribute significantly to environmental degradation and can have long-lasting climatic consequences. Belcher et al. (2020) point to high greenhouse gas emissions associated with military activities. Austin and Bruch (2000), in their comprehensive work on the environmental consequences of war, discuss a wide range of long-term impacts, including ecosystem destruction, resource depletion, and the potential for regional climatic changes due to large-scale environmental degradation. Providing a specific example, Husain (1998) analyzes the effects of the Gulf War, highlighting the impact of oil well fires and oil spills on local ecosystems and regional climate.

While the physical interplay between climate change and wars has been extensively studied, there is a notable lack of research examining how war experiences shape individuals’ perceptions and attitudes toward climate change. This gap in the literature is particularly significant when considering how people prioritize environmental concerns in the context of armed conflict. An exception to this scarcity is the study by Rinscheid and Koos (2023), who examined the impact of the war in Ukraine on Germans’ willingness to support climate actions. Their experiment showed no decrease in this willingness in the face of war. However, it is worth noting that this study was conducted in Germany, a country not directly involved in the conflict, and focused on support for climate actions rather than general climate change perceptions.

Despite the extensive research on the finite pool of worry in various crisis contexts, studies on natural hazards experiences and climate attitudes, and the established links between war and environmental degradation, a significant gap remains in our understanding. Specifically, there is a lack of research examining how individuals directly affected by war perceive and prioritize climate change concerns. This gap is particularly notable given the potential for armed conflicts to dramatically alter individuals’ immediate priorities and risk perceptions. While studies have explored how economic crises, pandemics, and natural hazards influence climate change attitudes, the unique context of war—with its immediate and severe threats to personal safety and societal stability—remains largely unexplored in relation to climate change perceptions. This gap in the literature limits our understanding of how extreme, conflict-driven circumstances might influence the perception and prioritization of long-term, global environmental threats.

Building on the FPW framework introduced earlier, this study proposes two competing hypotheses that reflect distinct theoretical perspectives on crisis response:

Primary hypothesis (FPW): The immediate and intense aftermath of war draws cognitive and emotional resources away from more distant threats, such as climate change, leading to decreased concern about long-term environmental issues among those more affected by the conflict.

Alternative hypothesis (generalized awareness): The experience of a major crisis like war increases overall risk awareness, including heightened concern for long-term environmental issues, even among those more affected by the conflict.

The first reflects a cognitive resource model, while the second draws on sociological and psychological theories—such as Beck’s risk society and Alexander’s cultural trauma—which emphasize sensitization to systemic risks and collective meaning-making.

Our study tests these competing predictions in a society experiencing direct warfare, asking: do individuals who perceive the war as having critically affected their lives show reduced or heightened concern about climate change?

According to the FPW hypothesis, individuals who have directly experienced war may allocate more of their limited capacity for worry to immediate and personal threats, potentially leading to a reduced allocation of concern for more distant, global threats like climate change. This shift in attention may result in a diminished perception of the urgency of climate risks and lower prioritization of environmental issues compared to immediate war-related concerns.

The study applies the FPW concept to the context of war experience and climate change perceptions, focusing on the unique case of the Russian–Ukrainian War. By examining how the perceived impact of war influences concerns about climate change among the Ukrainian population, it addresses an important empirical question about how acute crises affect perceptions of long-term global risks.

## Materials and Methods

The principal aim of this study is to explore the relationship between Ukrainians’ perceptions of how the war had impacted their lives and their concerns about climate change-related risks. To address this objective, a quantitative approach based on survey methodology was adopted. The research design, including the formulation of survey questions, was developed by the SOR-WAR Project research team. The field implementation of the survey was carried out as part of the “Opinions and Views of the Population of Ukraine” Omnibus study, conducted by the Kyiv International Institute of Sociology (KIIS) in September–October 2023.

A cross-sectional design was employed using computer-assisted telephone interviews (CATI) to collect data from a random sample of 2014 adult Ukrainian residents (aged 18 and older). To ensure representativeness, the sample was stratified by region and type of settlement (urban/rural), with post-stratification weights applied based on age, gender, and education to align with the demographic structure of the Ukrainian adult population.

Respondents were selected through random generation of mobile phone numbers, proportionally representing major mobile operators in Ukraine. The survey covered all regions of Ukraine under government control, excluding the Autonomous Republic of Crimea and territories not controlled by Ukrainian authorities prior to February 24, 2022. Thus, territories under Russian occupation were not included in the sampling frame.

Data collection occurred in 19–20 months into the full-scale invasion. While this period saw continued military operations and infrastructure attacks, these reflected the ongoing wartime situation rather than exceptional circumstances. Climate-wise, Ukraine experienced ongoing drought and above-average October temperatures, which could potentially heighten climate awareness rather than diminish it. We found no evidence of unusual events during the survey period that would systematically bias respondents’ climate change perceptions beyond the general wartime context already central to our study.

The analysis focuses on two key variables developed by the SOR-WAR Project team:

(1) Dependent Variable: Concerns about Climate Change This variable was measured as part of a Likert-type scale assessing perceptions of various global threats. Respondents were asked: “How worried are you about personally experiencing adverse weather phenomena related to climate change in the next 3 years?” The original five-point scale responses were recategorized into three levels:

- Low concern (original responses “Not at all worried” and “Rather not worried”)
- Moderate concern (original response “Somewhat worried, somewhat not”)
- High concern (original responses “Rather worried” and “Very worried”)

(2) Independent Variable: Perceived Impact of War This variable was assessed with the question: “Considering various aspects, please evaluate what impact the outbreak of war had on your life?” Responses were measured on an 11-point scale, where 0 meant “no impact at all” and 10 meant “my life changed completely”. For the analysis, these responses were recategorized into three levels:

- Relatively low impact (original responses 0–5)
- Significant impact (original responses 6–9)
- Critical impact (original response 10)

These variables were designed to capture the unique context of the ongoing conflict in Ukraine and its potential relationship with perceptions of long-term environmental risks.

Control variables included gender, age category, place of residence (urban/rural), and macroregion to account for potential confounding factors. These demographic and geographic factors were selected as they are known to influence both risk perceptions and exposure to war impacts. Gender and age consistently predict environmental concern, with women and younger individuals typically expressing higher climate change worry (Poortinga et al. 2019). Urban/rural residence affects both environmental awareness and war exposure patterns (Marquart-Pyatt 2012), while regional variation captures differential conflict intensity (Dodds et al. 2023) and historical–cultural divisions between regions (Kulyk 2016). We deliberately limited controls to these fundamental demographic and geographic variables to avoid overadjustment bias that could arise from including potential mediators of the war–climate concern relationship, such as economic status or displacement experience, which may themselves be consequences of war impact.  Table 1 presents the distributions of all study variables.

**Table 1:** Frequency distributions of study variables (*N* = 2014)

Variable	Scale/categories	*n*	%
Climate change concerns	1—Not at all worried	453	22.5
(5-point scale)	2—Rather not worried	210	10.4
	3—Somewhat worried, somewhat not	367	18.2
	4—Rather worried	393	19.5
	5—Very worried	591	29.4
War impact on life	0—No impact at all	41	2.0
(0–10 scale)	1	6	0.3
	2	8	0.4
	3	14	0.7
	4	14	0.7
	5—Medium impact	361	17.9
	6	60	3.0
	7	159	7.9
	8	207	10.3
	9	93	4.6
	10—Life changed completely	1052	52.2
Gender	Male	912	45.3
	Female	1102	54.7
Age groups	18–29	272	13.5
	30–39	453	22.5
	40–49	371	18.4
	50–59	334	16.6
	60–69	312	15.5
	70 +	273	13.5
Residence	Urban	1333	66.2
	Rural	681	33.8
Region	West	547	27.2
	Central	700	34.8
	South	497	24.7
	East	270	13.4

Multinomial logistic regression was employed using IBM SPSS Statistics 29.0. This method was chosen due to the categorical nature of the dependent variable, which was divided into three ordered levels of climate change concern. The model included the perceived impact of war as the primary independent variable, while adjusting for demographic and geographic factors.

This approach allows for the assessment of how the perceived impact of war influences climate change concerns while accounting for key demographic and geographic factors. By including these control variables, potential differences in war impact perception and climate change concerns across various groups and regions are considered. This method increases the accuracy of estimates by controlling regional and demographic variations in conflict exposure and their effects on both war experiences and climate perceptions.

The study was conducted in full compliance with ethical standards for social science research. The research protocol was reviewed and approved by the Ethics Committee of the Faculty Social Science at the Universty of Wroclaw. Data collection was carried out by a professional survey company following ESOMAR Code on Market, Opinion and Social Research and Data Analytics, which includes standard protocols for participant protection in sensitive contexts. These protocols ensure: informed consent, voluntary participation, right to withdraw at any time, anonymity of responses, and secure data handling. The telephone survey methodology allowed participants to respond from locations they deemed safe, minimizing potential risks associated with wartime conditions. The research team received only anonymized data without any personal identifiers. The dataset has been made publicly available in the Unversity of Wroclaw Repository with full anonymization maintained.^1^

## Results

This section highlights the key findings that address our central research question: How does the perceived impact of the ongoing war in Ukraine influence individuals’ concerns about climate change? Our analysis explores whether those more severely affected by the war exhibit decreased concern about climate change, or if experiencing one crisis might heighten awareness of other potential threats.

While detailed statistical analyses, procedures, and comprehensive results are available in the 10.1007/s13280-025-02291-w, this section zeroes in on the key findings related to our research question. It begins by presenting the distributions of key variables under study, providing essential context for understanding the diverse experiences and perceptions within our sample.

Data reveal that respondents expressed considerable concern about weather phenomena associated with climate change. Nearly half of the participants reported significant worry, with almost 30% stating they were greatly bothered by this issue, and an additional 20% indicating they were rather bothered. This high level of concern was not universal, however, as nearly a quarter of respondents (23%) reported little to no worry about climate change-related weather events. The remaining participants, comprising about 18% of the sample, expressed a neutral stance, being neither particularly bothered nor unbothered by these phenomena.

For analytical purposes, these responses were categorized into three levels of concern. High concern, encompassing those greatly or rather bothered, represented 48.9% of the sample. Low concern, including those rather not bothered or not bothered at all, accounted for 32.9%. The neutral responses were classified as moderate concern, making up 18.2% of participants.

The war’s impact on respondents’ lives was profound and widespread, as evidenced by the descriptive statistics. On a scale from 0 (no impact) to 10 (complete life change), the mean perceived impact of war was 8.15, with a mode of 10, indicating an extremely high and skewed impact distribution. The standard deviation of 2.41 reflects considerable variation in responses, albeit predominantly in the upper range of the impact scale. Notably, very few respondents reported low impact levels; only 4.1% indicated scores between 0 and 4, underscoring the pervasive effect of the conflict across the population.

For analytical purposes, responses regarding war impact were categorized into three levels to reflect the distribution of the variable. Critical impact, representing those who reported the maximum score of 10, encompassed 52.2% of respondents. Significant impact, covering scores from 6 to 9, accounted for 25.8% of the sample. The remaining 22.0% of respondents, reporting scores from 0 to 5, were classified as experiencing relatively low impact.

To analyze the relationship between the perceived impact of war and climate change concerns, we employed multinomial logistic regression. This method allows us to examine how different levels of war impact influence the likelihood of expressing various degrees of climate change concern while simultaneously controlling for other relevant factors. The model’s overall fit was significant (*χ*^2^ = 183.476, df = 24, *p* < 0.001), indicating that it performs better than a null model. The Nagelkerke pseudo *R*-square value of 0.100 suggests that the model explains approximately 10% of the variance in climate change concerns.

Table 2 presents the key results of the multinomial logistic regression. The coefficients (*B*) represent the change in log-odds of being in the respective category of climate change concern (moderate or high) compared to the reference category (low concern) for each level of war impact. Positive values indicate an increase in the likelihood of the outcome. The odds ratios (Exp(*B*)) are derived from these coefficients and indicate how many times more likely an outcome is for each level of war impact compared to the reference category (relatively low impact). A value greater than 1 indicates increased odds, while a value less than 1 indicates decreased odds. The 95% confidence intervals (CI) provide a range within which we can be 95% certain the true population value lies. The *p* values indicate the statistical significance of the results, with values below 0.05 generally considered significant.

**Table 2:** Multinomial logistic regression results: war impact and control variables predicting climate change concerns

	*n* (*N*)	*B*	Exp(*B*)	95% CI	*p* value
*MODERATE CLIMATE CONCERNS (ref: low concerns)*					
War impact (ref: relatively low)	80 (444)	–	1 (ref.)	–	–
Significant	97 (518)	0.124	1.132	[0.778, 1.648]	0.517
Critical	190 (1052)	0.428	1.535	[1.094, 2.153]	**0.013**
Gender (ref: female)	209 (1101)	–	1 (ref.)	–	–
Male	158 (913)	− 0.466	0.628	[0.483, 0.815]	**< 0.001**
Age (ref: 70+)	41 (273)	–	1 (ref.)	–	–
18–29	48 (272)	− 0.463	0.630	[0.370, 1.071]	0.088
30–39	85 (453)	− 0.142	0.868	[0.535, 1.408]	0.566
40–49	81 (370)	− 0.094	0.910	[0.558, 1.484]	0.706
50–59	63 (335)	0.061	1.063	[0.635, 1.779]	0.817
60–69	49 (311)	0.080	1.083	[0.631, 1.858]	0.772
Urban/rural (ref: rural)	116 (681)	–	1 (ref.)	–	–
Urban	251 (1334)	− 0.020	0.980	[0.735, 1.306]	0.890
Region (ref: East)	55 (270)	–	1 (ref.)	–	–
West	81 (546)	− 0.042	0.959	[0.617, 1.491]	0.852
Central	135 (700)	0.308	1.361	[0.910, 2.035]	0.134
South	95 (496)	0.221	1.247	[0.819, 1.898]	0.304
*HIGH CLIMATE CONCERNS (ref: low concerns)*					
War impact (ref: relatively low)	194 (444)	–	1 (ref.)	–	–
Significant	206 (518)	0.244	1.277	[0.943, 1.728]	0.114
Critical	584 (1052)	0.856	2.353	[1.797, 3.080]	**< 0.001**
Gender (ref: female)	594 (1101)	–	1 (ref.)	–	–
Male	390 (913)	− 0.557	0.573	[0.465, 0.706]	**< 0.001**
Age (ref: 70+)	168 (273)	–	1 (ref.)	–	–
18–29	90 (272)	− 1.231	0.292	[0.193, 0.442]	**< 0.001**
30–39	211 (453)	− 0.709	0.492	[0.340, 0.712]	**< 0.001**
40–49	147 (370)	− 0.945	0.389	[0.265, 0.571]	**< 0.001**
50–59	178 (335)	− 0.361	0.697	[0.469, 1.034]	0.073
60–69	190 (311)	− 0.006	0.994	[0.662, 1.492]	0.976
Urban/rural (ref: rural)	355 (681)	–	1 (ref.)	–	–
Urban	630 (1334)	− 0.115	0.891	[0.711, 1.117]	0.317
Region (ref: East)	109 (270)	–	1 (ref.)	–	–
West	278 (546)	0.615	1.850	[1.297, 2.639]	**< 0.001**
Central	351 (700)	0.671	1.957	[1.398, 2.739]	**< 0.001**
South	245 (496)	0.524	1.689	[1.190, 2.398]	**0.003**

Model fit: *χ*^2^ = 183.476, df = 24, *p* < .001; Nagelkerke *R*^2^ = 0.100

*n* = number of respondents in category with moderate/high climate concern; (*N*) = total respondents in category. Significant *p* values (*p* < .05) shown in bold

The results reveal that critical experiences of war significantly increase the likelihood of expressing higher concerns about weather and climate-related issues. Individuals who reported a critical impact of war were 2.353 times more likely to express high concerns about climate change compared to those who reported a relatively low impact (*B* = 0.856, Exp(*B*) = 2.353, 95% CI [1.797, 3.080], *p* < 0.001). This relationship was also present, albeit weaker, for moderate climate change concerns, with those reporting critical war impact being 1.535 times more likely to express moderate concerns (*B* = 0.428, Exp(*B*) = 1.535, 95% CI [1.094, 2.153], *p* = 0.013).

Interestingly, substantial (but not critical) impact of war did not show a statistically significant relationship with either moderate or high climate change concerns. This suggests that only the most severe experiences of war are associated with increased climate change concerns.

To verify the robustness of these findings, we conducted an additional analysis using ordinal logistic regression. The test of parallel lines (*p* = 0.511) confirmed the appropriateness of this approach, and the results remained highly consistent with the multinomial model. Critical war impact maintained its strong positive effect on climate change concerns (*b* = 0.722, *p* < 0.001), while substantial war impact remained nonsignificant (*p* = 0.082). Complete results from both analytical approaches are provided in the 10.1007/s13280-025-02291-w.

The model also accounted for several control variables. Gender, age, and region of residence were found to be significant predictors of climate change concerns, while urban/rural residence did not show a significant effect. Women were more likely than men to express high climate change concerns, and younger respondents were more likely to report high concerns compared to older age groups. Regional differences were also observed, with respondents from Western and Central regions more likely to express high concerns about climate change compared to those from the East. Specifically, respondents from Western regions were 1.85 times more likely (*p* < 0.001) and those from Central regions 1.96 times more likely (*p* < 0.001) to express high climate concerns compared to Eastern regions. This regional variation is noteworthy. However, these regional differences do not negate our main finding: the positive relationship between critical war impact and climate concern remains significant even after controlling for region. This suggests that while baseline climate concerns vary by region—possibly reflecting historical, cultural, and economic differences—the sensitizing effect of severe war experiences on environmental awareness operates across all regions.

## Discussion

The main findings of our study provide interesting insights into the relationship between the experience of war and concern about climate change. They indicate that those experiencing the critical impact of war are significantly more likely to express high levels of concern about climate change compared to those who experienced a relatively low impact conflict. This result stands in opposition to the original FPW hypothesis proposed by Weber (2006), which suggested that direct experience of one crisis may reduce the ability to worry about other, more distant threats. Instead, our results seem to support the alternative effect generalization hypothesis, according to which the experience of one threat can sensitize individuals to other potential risks.

These observations align with some of the most recent research that also questions the universality of FPW. For instance, Sisco et al. (2023) in their comprehensive study on the COVID-19 pandemic observed that while attention to climate change decreased, the level of worry about this phenomenon did not diminish, and in some cases even increased. Similarly, the current findings correspond with those of Stefkovics et al. (2024), who noted an increase in climate change concerns across several European countries during the COVID-19 pandemic.

It is worth noting, however, that these findings do not entirely negate the concept of FPW. It is possible that a complex interaction between different psychological mechanisms is being observed. For example, while FPW may operate in the short term or in cases of less intense experiences, prolonged or particularly intense exposure to a threat may lead to a generalization of concerns, as the current results suggest.

Several psychological mechanisms may explain why individuals reporting critical war impact exhibit heightened climate concerns. First, trauma may sensitize individuals to systemic threats, as it fundamentally undermines basic assumptions of safety and control (Janoff-Bulman 1992). Second, witnessing infrastructure collapse may increase awareness of systemic vulnerability, making climate disruption more imaginable. Third, war often brings people closer to questions of mortality and legacy, intensifying concerns for future generations and environmental stability.

Importantly, this effect appears only among those reporting a critical war impact, suggesting a threshold mechanism: only severe crisis experiences are strong enough to trigger cognitive reorganization and heightened environmental concern. Moderate exposure may not overcome the psychological distance often associated with climate change, failing to activate the sensitization processes described above.

However, moving beyond strictly psychological interpretations of the results obtained, it is worth considering the broader, sociological dimension of the phenomena observed. Such an approach makes it possible to situate individual reactions and concerns in a broader social context, taking into account the complex interactions between different crises and their impact on collective threat perceptions.

In the context of the Russian–Ukrainian conflict, the phenomenon of environmental terrorism, which involves the intentional impact on the natural environment in order to achieve tactical or strategic military advantage, assumes particular importance. For example, there have been concerns in public discourse about the possible triggering of environmental catastrophes, such as the crisis at the Zaporizhzhia Nuclear Power Plant (Russia Approves Plan to Blow up Zaporizhzhia Nuclear Power Plant 2023), blowing up of the Novaya Kakhovka dam (PociянипiдipвaлигpeблюКaxoвcькoïГEC 2023), or Russian provocations related to the use of chemical weapons (The Russian Federation Started Implementing Provocations with Chemical Weapons 2023). In addition, concern was expressed about the possible escalation of the conflict, which could put additional strain on the environment, especially if nuclear weapons were used (Izhak 2022).

The increased concern about climate change among those most affected by the war, which was observed in the referenced survey, may reflect an awareness of these risks. For example Saxena (2023) highlights how warfare operations have an irreversible and massive impact on greenhouse gas concentrations, suspended particulate matter and ecological footprints, contributing to climate change. Ali et al. (2023) at theirs part, show how conflict affects energy markets and consumption patterns in Europe, potentially leading to increased use of fossil fuels in some countries, with important implications for climate change mitigation efforts.

These concerns correspond with expert reports and scientific analyses that point to significant environmental and climate risks as a consequence of ongoing war. For instance, Kicaj et al. (2023) highlight that the climate impacts of warfare in Ukraine may extend beyond the immediate conflict zone, which corresponds with respondents’ concerns about the wider environmental consequences of war. Solokha et al. (2023) provide evidence of direct environmental damage caused by the conflict, such as increased heavy metal content in soils in areas under fire, which may translate into increased environmental awareness among those experiencing war.

While the study results challenge the FPW hypothesis in this specific context, they highlight the complex social processes involved in risk perception and collective sense-making. The findings suggest that severe societal disruptions, such as war, can lead to a broader reevaluation of risks, including those that may have previously seemed distant or abstract.

These findings can be interpreted through the lens of Ulrich Beck's (1992)“risk society” theory and its developments (Rosa et al. 2014). Beck posits that modern societies are increasingly preoccupied with debating, preventing, and managing risks that are often global in nature and transcend traditional social boundaries. The heightened concern about climate change among those most affected by the war may indicate a broader shift toward a "risk consciousness" in Ukrainian society. As Beck argues, experiences of manufactured risks (in this case, war) can heighten awareness of other, less immediately tangible risks (such as climate change). This interpretation aligns with the observed results, where those experiencing critical war impacts show increased, rather than decreased, concern for climate issues. This is not a simple transfer of anxiety from one risk to another, but rather the result of a deeper understanding of the interconnectedness of different risks in the modern world.

This interpretation can be further enriched by Alexander’s (2004) theory of cultural trauma, which complements Beck’s risk society framework by emphasizing the collective processes of meaning-making that emerge in response to catastrophic events. Rather than viewing heightened climate concern among war-affected individuals as a mere psychological reaction, Alexander’s perspective suggests that such responses reflect a deeper cultural reconfiguration of systemic vulnerabilities. In this light, war experiences may not only heighten awareness of specific risks but also deepen the understanding that environmental and geopolitical instabilities reflect interconnected expressions of the same underlying systemic fragilities. This view is also supported by Hirst et al. (2020), who show that cultural trauma can arise from shared emotional memories of public events, reinforcing collective awareness of broader systemic threats.

Such perspective aligns with the concept of polycrisis (Henig and Knight 2023), which examines how multiple crises can interact and amplify each other. In this framework, the war in Ukraine can be viewed not as an isolated event, but as part of a complex of interconnected crises that include climate change. This approach situates the conflict in Ukraine as both a consequence and a cause of broader global tensions and problems, including climate and ecological issues.

Reference can be made here to Ulrich Beck’s concept of “reflexive modernization” (Beck 1992), which suggests that as societies become more aware of the threats they face, they also become more reflexive about these threats and their interconnections. When viewed in this way, the results obtained can be interpreted as a form of social reflexivity, although driven by academic or expert discourse, is also increasingly apparent in the public consciousness. Ukrainian respondents, to a large extent, seem to interpret the war not only as an international conflict, but as a manifestation of broader global tensions and system invulnerabilities. Their perception transcends individual experiences, encompassing a more comprehensive understanding of the war’s implications. They view the conflict as a catalyst for fundamental macrosocial changes with far-reaching global consequences. This expanded perspective integrates various dimensions of the crisis, including its environmental and climatic ramifications. Respondents who perceive the war as having a critical impact on their lives may do so not only because of its immediate effects, but because they recognize its potential to reshape global systems, including environmental and climate dynamics.

In other words, the observed link between the perception that war critically alters the lives of respondents and increased environmental and climate concerns reflects a functioning conviction in the social consciousness of “synchronous failure.” Homer-Dixon et al. (2015), i.e., the cascading collapse of successive, crisis-affected spheres of modern civilization.

The model’s explanatory power of around 10% of the variance in climate change concerns indicates that while perceptions of the impact of war are significant, it is not the only, or perhaps even the most important, factor shaping these concerns. This observation is consistent with other studies in environmental sociology, which typically find that environmental attitudes are influenced by multiple interrelated factors (Hornsey et al. 2016).

These findings suggest that climate concern can persist even during severe conflict, providing a potential foundation for environmental peacebuilding (Ide 2020). Shared environmental risks may serve as a platform for post-conflict dialogue and cooperation, as seen in proposals for "green recovery" in Ukraine. Research-confirmed awareness of the interconnectedness of conflicts and environmental problems can underpin such initiatives, laying the groundwork for cooperation even during ongoing conflict. This environmental focus creates space for dialogue in areas not directly related to the dispute itself, potentially engaging third parties such as the United States, the European Union, or China in strengthening regional peace and stability.

However, the practical application of the “environmental peacebuilding” approach in the context of the Russian–Ukrainian war appears to be exposed to serious challenges and obstacles familiar from other conflicts (Ide et al. 2021; Krampe 2021). Nevertheless, while environmental hazards are unlikely to become a key element and cause of direct peacebuilding efforts, they can serve as a backdrop and natural platform for dialogue in both conflict resolution and post-conflict reconstruction policies (Swain and Öjendal 2018).

## Conclusions

This study investigated the relationship between the perceived impact of the Russian–Ukrainian war and concerns about climate change among Ukrainian citizens. It aimed to test competing hypotheses about how crisis experiences affect perceptions of long-term environmental threats during an ongoing armed conflict. The research provides insights into how societies balance immediate crisis threats with long-term environmental risks.

The results reveal a significant positive relationship between critical war impact and increased climate change concerns. Individuals who reported that the war had a critical impact on their lives were more likely to express high levels of concern about climate change compared to those less affected. This finding challenges the “finite pool of worry” hypothesis, which suggests that immediate crises might reduce concern for more distant threats. Instead, it indicates that severe crisis experiences may heighten awareness of other potential risks and long-term challenges.

These findings contribute to a broader understanding of risk perception in societies dealing with multiple crises. They point to a complex social dynamic that goes beyond the simplistic opposition between the “finite pool of worry” and generalization effect hypotheses. The increased climate change concerns among those critically affected by war suggest that societies experiencing severe crises may develop a heightened awareness of interconnected global risks. This aligns with sociological perspectives on “risk society” and “polycrisis”, where dealing with multiple, interrelated risks becomes a permanent state. The study thus argues for understanding risk perception and prioritization in crisis contexts as a broader social process, moving beyond individual psychological explanations to consider how societies collectively navigate interconnected threats.

The study has several limitations. The cross-sectional design limits our ability to establish causality or track changes over time and may be influenced by idiosyncratic confounders. The lack of prewar data makes it challenging to determine if findings reflect war-induced changes or preexisting attitudes. Data collection during ongoing drought and above-average temperatures could have heightened baseline climate awareness, though we found no unusual events that would systematically bias responses beyond the general wartime context. Additional limitations include reliance on self-reported data, potential oversimplification through response categorization, incomplete exploration of underlying mechanisms, and limited generalizability beyond the Ukrainian context. An open question remains whether respondents distinguish environmental destruction caused by war from anthropogenic climate change, or whether these are cognitively conflated during crisis. However, for our central finding—that severe crisis experiences heighten rather than diminish environmental concern: the specific sources of perceived threat may be secondary. Future research should explore these mechanisms through longitudinal studies tracking perception changes over time, comparative analyses across conflict zones, and in-depth investigations of how war experiences translate into environmental attitudes and behaviors.

The study’s insights may have implications for environmental peacebuilding. The persistence of climate concerns amid severe conflict experiences challenges the notion that immediate crises necessarily diminish attention to long-term environmental threats. While environmental issues may not be a primary focus in the Russian–Ukrainian conflict, this environmental awareness could potentially inform post-conflict dialogue and reconstruction efforts. However, further research is needed to explore how these concerns might translate into practical peacebuilding approaches. These results suggest that environmental initiatives may be more crisis-resilient than previously assumed, with potential implications for climate policy during conflicts and postcrisis reconstruction efforts.

## Supplementary Information

Below is the link to the electronic supplementary material.

Supplementary file1 (PDF 521 KB)

## Data Availability

The data that support the findings of this study are openly available in the University of Wrocław Repository at https://repozytorium.uni.wroc.pl/en/dlibra/publication/150096.

## Footnotes

1 Available at http://repozytorium.uni.wroc.pl/en/dlibra/publication/150096.
