# Supplementary material for: Beyond the finite pool of worry: War experiences and climate change concerns in Ukraine
Source: Ambio. 2025 Nov 15;55(4):844–56. doi: 10.1007/s13280-025-02291-w (PMC12960963; doi:10.1007/s13280-025-02291-w)
Supplement: Supplementary file 1 — Supplementary file1 (PDF 521 KB) [file 13280_2025_2291_MOESM1_ESM.pdf]

SUPPLEMENTARY MATERIALS

Statistical Appendix for "Beyond the Finite Pool of Worry: War Experiences and Climate Change Concerns in Ukraine"

This document contains the results of the statistical analyses carried out, including: the distributions of the variables studied, the procedures for transforming the variables and the distributions of the transformed variables, the cross-table analysis of the variables studied, and the analysis of the nominal regression model. The analyses were performed in IBM SPSS v. 29.0. To ensure transparency, the complete SPSS syntax used to conduct the statistical analysis is included.

VARIABLES UNDER STUDY

INVESTIGATED VARIABLES

A. Assessment of the impact of war on the respondent's life

Survey question: *Tell me, please, how the war between Russia and Ukraine affected your life? Rate on a scale of 0 to 10, where 0 means no impact at all, 5 means a medium impact, and 10 means my life has completely changed.*

Name of the variable in dataset: **Zh2**

Distribution

FREQUENCIES VARIABLES=Zh2

/ORDER=ANALYSIS.

Tell me, please, how the war between Russia and Ukraine affected your life? Rate on a scale of 0 to 10, where 0 means no impact at all, 5 means a medium impact, and 10 means my life has completely changed.

VARIABLE NAME: ***Zh2***

	N	%
0 - It had no impact at all	41	2,0%

1	6	0,3%
2	8	0,4%
3	14	0,7%
4	14	0,7%
5 - A medium impact	361	17,9%
6	60	3,0%
7	159	7,9%
8	207	10,3%
9	93	4,6%
10 - My life has completely changed	1052	52,2%

```
EXAMINE VARIABLES=Zh2
  /PLOT NONE
  /STATISTICS DESCRIPTIVES
  /CINTERVAL 95
  /MISSING LISTWISE
  /NOTOTAL.
```

Descriptives

**Zh2**		Statistic	Std. Error
	Mean	8,1465	,05362

Tell me, please, how the war between Russia and Ukraine affected your life? Rate on a scale of 0 to 10, where 0 means no impact at all, 5 means a medium impact, and 10 means my life has completely changed.	95% Confidence Interval for Mean	Lower Bound	8,0414	
		Upper Bound	8,2517	
	5% Trimmed Mean		8,3798	
	Median		10,0000	
	Variance		5,790	
	Std. Deviation		2,40622	
	Minimum		0	
	Maximum		10	
	Range		10	
	Interquartile Range		3,62	
	Skewness		-1,221	,055
	Kurtosis		,997	,109

Variable to be recoded:

```
RECODE Zh2 (10=0) (6 thru 9=1) (0 thru 5=2) INTO WAR_IMPACT2.
EXECUTE.
```

Name of the output variable: **WAR_IMPACT2 Impact of war assessment**

Output variable distribution:

```
FREQUENCIES VARIABLES=WAR_IMPACT2
  /ORDER=ANALYSIS.
```

Impact of war assessment

	N	%
0 critical impact	1052	52,2%
1 significant impact	519	25,8%
2 relatvely low impact	443	22,0%

B. Concerns about climate change

Survey question: *How worried are you about a possibility that the following events may happen to you within the next 3 years?*

Please rate your concern on a scale where 1 means it doesn't bother me at all, 2 means it rather doesn't bother me, 3 means it bothers me a little, and doesn't bother me little, 4 means it rather bothers me, and 5 means it bothers me a lot.

Name of the variable in dataset: ***Zh3_6*** Adverse weather phenomena associated with climate change

DISTRIBUTION

```
FREQUENCIES VARIABLES=Zh3_6

  /ORDER=ANALYSIS.
```

Adverse weather phenomena associated with climate change

	N	%
1,00 It doesn't bother me at all	453	22,5%

2,00 It rather doesn't bother me	210	10,4%
3,00 It bothers me a little, and doesn't bother me little	367	18,2%
4,00 It rather bothers me	393	19,5%
5,00 It bothers me a lot	591	29,4%

Variable to be recoded:

RECODE Zh3_6 (3=1) (1 thru 2=0) (4 thru 5=2) INTO CLIMAT_CHANGE.

VARIABLE LABELS CLIMAT_CHANGE 'Concerns about climate change'.

EXECUTE.

Output variable: **CLIMAT_CHANGE Concerns about climate change**

Output variable distribution:

FREQUENCIES VARIABLES=CLIMAT_CHANGE

/ORDER=ANALYSIS.

CLIMAT_CHANGE Concerns about climate change

	N	%
0 low	663	32,9%
1 moderate	367	18,2%

2 high	984	48,9%

SOCIO-DEMOGRAPHIC CHARACTERISTICS

FREQUENCIES VARIABLES=sd1 age_6gr urban_rural region4

/ORDER=ANALYSIS.

gender SD1. Gender:

	N	%
1,00 Male	912	45,3%
2,00 Female	1102	54,7%

age Age (6 age groups)

	N	%
1,00 18-29	272	13,5%
2,00 30-39	453	22,5%
3,00 40-49	371	18,4%
4,00 50-59	334	16,6%
5,00 60-69	312	15,5%
6,00 70+	273	13,5%

urban_rural Urban and rural population

	N	%
1,00 Urban	1333	66,2%
2,00 Rural	681	33,8%

region4 4 Regions

	N	%
1,00 West	547	27,2%
2,00 Central	700	34,8%
3,00 South	497	24,7%
4,00 East	270	13,4%

ANALYSIS

Contingency table analysis

```
CROSSTABS
  /TABLES=CLIMAT_CHANGE BY WAR_IMPACT2
  /FORMAT=AVALUE TABLES
```

/STATISTICS=CHISQ

/CELLS=COUNT COLUMN

/COUNT ROUND CELL.

CLIMAT_CHANGE Concerns about climate change * WAR_IMPACT2 Impact of war assesment Crosstabulation

		WAR_IMPACT2 Impact of war assesment							
		,00 critical impact		1,00 significant impact		2,00 relatvely low impact		Total	
		N	%	N	%	N	%	N	%
CLIMAT_CHANGE Concerns about climate change	,00 low	278	26,4%	215	41,5%	170	38,3%	663	32,9%
	1,00 modderate	190	18,1%	97	18,7%	80	18,0%	367	18,2%
	2,00 high	584	55,5%	206	39,8%	194	43,7%	984	48,9%
Total		1052	100,0%	518	100,0%	444	100,0%	2014	100,0%

Chi-Square Tests

	Value	df	Asymptotic Significance (2-sided)
Pearson Chi-Square	49,781[a]	4	<,001
Likelihood Ratio	49,961	4	<,001
Linear-by-Linear Association	32,927	1	<,001

N of Valid Cases	2014		

a. 0 cells (0,0%) have expected count less than 5. The minimum expected count is 80,91.

Multinominal Logistic Regression

```
NOMREG CLIMAT_CHANGE (BASE=FIRST ORDER=ASCENDING) BY WAR_IMPACT2 sd1 age_6gr urban_rural region4
  /CRITERIA CIN(95) DELTA(0) MXITER(100) MXSTEP(5) CHKSEP(20) LCONVERGE(0) PCONVERGE(0.000001)
    SINGULAR(0.00000001)
  /MODEL
  /STEPWISE=PIN(.05) POUT(0.1) MINEFFECT(0) RULE(SINGLE) ENTRYMETHOD(LR) REMOVALMETHOD(LR)
  /INTERCEPT=INCLUDE
  /PRINT=PARAMETER SUMMARY LRT CPS STEP MFI.
```

Case Processing Summary

		N	Marginal Percentage
CLIMAT_CHANGE Concerns about climate change	,00 low	663,08	32,9%
	1,00 modderate	366,76	18,2%
	2,00 high	984,16	48,9%
WAR_IMPACT2 Impact of war assesment	,00 critical impact	1051,87	52,2%
	1,00 significant impact	518,73	25,8%

	2,00 relatvely low impact	443,40	22,0%
sd1 SD1. Gender:	1,00 Male	912,43	45,3%
	2,00 Female	1101,57	54,7%
age_6gr Age (6 age groups)	1,00 18-29	271,97	13,5%
	2,00 30-39	452,64	22,5%
	3,00 40-49	370,67	18,4%
	4,00 50-59	334,26	16,6%
	5,00 60-69	311,86	15,5%
	6,00 70+	272,59	13,5%
urban_rural Urban and rural population	1,00 Urban	1333,02	66,2%
	2,00 Rural	680,98	33,8%
region4 4 Regions	1,00 West	546,89	27,2%
	2,00 Central	700,29	34,8%
	3,00 South	496,85	24,7%
	4,00 East	269,96	13,4%
Valid		2014,00	100,0%
Missing		,00	
Total		2014,00	
Subpopulation		242[a]	

a. The dependent variable has only one value observed in 64 (26,4%) subpopulations.

Model Fitting Information

Model	Model Fitting Criteria	Likelihood Ratio Tests		
	-2 Log Likelihood	Chi-Square	df	Sig.
Intercept Only	1737,358			
Final	1553,882	183,476	24	<,001

Pseudo R-Square

Cox and Snell	,087
Nagelkerke	,100
McFadden	,044

Likelihood Ratio Tests

Effect	Model Fitting Criteria	Likelihood Ratio Tests		
	-2 Log Likelihood of Reduced Model	Chi-Square	df	Sig.

Intercept	1553,882[a]	,000	0	.
WAR_IMPACT2 Impact of war assesment	1601,715	47,833	4	<,001
sd1 SD1. Gender:	1582,853	28,971	2	<,001
age_6gr Age (6 age groups)	1626,384	72,502	10	<,001
urban_rural Urban and rural population	1555,035	1,153	2	,562
region4 4 Regions	1575,676	21,794	6	,001

The chi-square statistic is the difference in -2 log-likelihoods between the final model and a reduced model. The reduced model is formed by omitting an effect from the final model. The null hypothesis is that all parameters of that effect are 0.

a. This reduced model is equivalent to the final model because omitting the effect does not increase the degrees of freedom.

Parameter Estimates

CLIMAT_CHANGE Concerns about climate change[a]		B	Std. Error	Wald	df	Sig.	Exp(B)	95% Confidence Interval for Exp(B) Lower Bound	Upper Bound
1,00 modderate	Intercept	-,627	,303	4,268	1	,039			
	[WAR_IMPACT2 Impact of war assesment=,00]	,428	,173	6,141	1	,013	1,535	1,094	2,153

	[WAR_IMPACT2 Impact of war assesment=1,00]	,124	,191	,421	1	,517	1,132	,778	1,648
	[WAR_IMPACT2 Impact of war assesment=2,00]	0[b]	.	.	0	.	.	.	.
	[sd1 SD1. Gender:=1,00]	-,466	,134	12,152	1	<,001	,628	,483	,815
	[sd1 SD1. Gender:=2,00]	0[b]	.	.	0	.	.	.	.
	[age_6gr Age (6 age groups)=1,00]	-,463	,271	2,917	1	,088	,630	,370	1,071
	[age_6gr Age (6 age groups)=2,00]	-,142	,247	,330	1	,566	,868	,535	1,408
	[age_6gr Age (6 age groups)=3,00]	-,094	,250	,142	1	,706	,910	,558	1,484
	[age_6gr Age (6 age groups)=4,00]	,061	,263	,054	1	,817	1,063	,635	1,779
	[age_6gr Age (6 age groups)=5,00]	,080	,275	,084	1	,772	1,083	,631	1,858
	[age_6gr Age (6 age groups)=6,00]	0[b]	.	.	0	.	.	.	.
	[urban_rural Urban and rural population=1,00]	-,020	,146	,019	1	,890	,980	,735	1,306
	[urban_rural Urban and rural population=2,00]	0[b]	.	.	0	.	.	.	.
	[region4 4 Regions=1,00]	-,042	,225	,035	1	,852	,959	,617	1,491

	[region4 4 Regions=2,00]	,308	,205	2,249	1	,134	1,361	,910	2,035
	[region4 4 Regions=3,00]	,221	,215	1,057	1	,304	1,247	,819	1,898
	[region4 4 Regions=4,00]	0[b]	.	.	0	.	.	.	.
2,00 high	Intercept	,278	,239	1,355	1	,244			
	[WAR_IMPACT2 Impact of war assesment=,00]	,856	,137	38,749	1	<,001	2,353	1,797	3,080
	[WAR_IMPACT2 Impact of war assesment=1,00]	,244	,154	2,504	1	,114	1,277	,943	1,728
	[WAR_IMPACT2 Impact of war assesment=2,00]	0[b]	.	.	0	.	.	.	.
	[sd1 SD1. Gender:=1,00]	-,557	,106	27,350	1	<,001	,573	,465	,706
	[sd1 SD1. Gender:=2,00]	0[b]	.	.	0	.	.	.	.
	[age_6gr Age (6 age groups)=1,00]	-1,231	,212	33,755	1	<,001	,292	,193	,442
	[age_6gr Age (6 age groups)=2,00]	-,709	,188	14,160	1	<,001	,492	,340	,712
	[age_6gr Age (6 age groups)=3,00]	-,945	,196	23,259	1	<,001	,389	,265	,571
	[age_6gr Age (6 age groups)=4,00]	-,361	,202	3,214	1	,073	,697	,469	1,034
	[age_6gr Age (6 age groups)=5,00]	-,006	,207	,001	1	,976	,994	,662	1,492

	[age_6gr Age (6 age groups)=6,00]	0[b]	.	.	0	.	.	.	.
	[urban_rural Urban and rural population=1,00]	-,115	,115	1,001	1	,317	,891	,711	1,117
	[urban_rural Urban and rural population=2,00]	0[b]	.	.	0	.	.	.	.
	[region4 4 Regions=1,00]	,615	,181	11,510	1	<,001	1,850	1,297	2,639
	[region4 4 Regions=2,00]	,671	,172	15,300	1	<,001	1,957	1,398	2,739
	[region4 4 Regions=3,00]	,524	,179	8,610	1	,003	1,689	1,190	2,398
	[region4 4 Regions=4,00]	0[b]	.	.	0	.	.	.	.

a. The reference category is: ,00 low.

b. This parameter is set to zero because it is redundant.

Ordinal Regression - PLUM

```
PLUM CLIMAT_CHANGE BY WAR_IMPACT2 gender age urban_rural region4
  /CRITERIA=CIN(95) DELTA(0) LCONVERGE(0) MXITER(100) MXSTEP(5) PCONVERGE(1.0E-6) SINGULAR(1.0E-8)
  /LINK=LOGIT
  /PRINT=FIT PARAMETER SUMMARY.
```

Case Processing Summary

		N	Marginal Percentage
Concerns about climate change	low	663,08	32,9%
	moderate	366,76	18,2%
	high	984,16	48,9%
Impact of war assesment	critical impact	1051,87	52,2%
	significant impact	518,73	25,8%
	relatvely low impact	443,40	22,0%
SD1. Gender:	Male	912,43	45,3%
	Female	1101,57	54,7%
Age (6 age groups)	18-29	271,97	13,5%
	30-39	452,64	22,5%
	40-49	370,67	18,4%
	50-59	334,26	16,6%
	60-69	311,86	15,5%
	70+	272,59	13,5%
Urban and rural population	Urban	1333,02	66,2%

	Rural	680,98	33,8%
4 Regions	West	546,89	27,2%
	Central	700,29	34,8%
	South	496,85	24,7%
	East	269,96	13,4%
Valid		2014,00	100,0%
Missing		,00	
Total		2014,00	

Model Fitting Information

Model	-2 Log Likelihood	Chi-Square	df	Sig.
Intercept Only	1737,358			
Final	1564,923	172,435	12	<,001

Link function: Logit.

Goodness-of-Fit

	Chi-Square	df	Sig.
Pearson	769,945	470	<,001

Deviance	883,977	470	<,001

Link function: Logit.

Pseudo R-Square

Cox and Snell	,082
Nagelkerke	,094
McFadden	,042

Link function: Logit.

Parameter Estimates

							95% Confidence Interval	
		Estimate	Std. Error	Wald	df	Sig.	Lower Bound	Upper Bound
Threshold	[CLIMAT_CHANGE = 0]	-,665	,198	11,284	1	<,001	-1,052	-,277
	[CLIMAT_CHANGE = 1]	,151	,197	,583	1	,445	-,236	,537
Location	[WAR_IMPACT2=0]	,722	,113	40,578	1	<,001	,500	,945
	[WAR_IMPACT2=1]	,223	,128	3,019	1	,082	-,029	,474
	[WAR_IMPACT2=2]	0[a]	.	.	0	.	.	.
	[gender=1,00]	-,446	,088	25,808	1	<,001	-,618	-,274

	[gender=2,00]	0[a]	.	.	0	.	.	.
	[age=1,00]	-1,088	,175	38,803	1	<,001	-1,430	-,745
	[age=2,00]	-,638	,154	17,087	1	<,001	-,941	-,336
	[age=3,00]	-,839	,160	27,519	1	<,001	-1,152	-,525
	[age=4,00]	-,359	,164	4,782	1	,029	-,680	-,037
	[age=5,00]	-,028	,168	,028	1	,867	-,358	,302
	[age=6,00]	0[a]	.	.	0	.	.	.
	[urban_rural=1,00]	-,090	,095	,892	1	,345	-,275	,096
	[urban_rural=2,00]	0[a]	.	.	0	.	.	.
	[region4=1,00]	,525	,150	12,324	1	<,001	,232	,819
	[region4=2,00]	,538	,140	14,673	1	<,001	,262	,813
	[region4=3,00]	,429	,146	8,588	1	,003	,142	,716
	[region4=4,00]	0[a]	.	.	0	.	.	.

Link function: Logit.

a. This parameter is set to zero because it is redundant.
